# The Kinetoplastid-Specific Protein TcCAL1 Plays Different Roles During *In Vitro* Differentiation and Host-Cell Invasion in *Trypanosoma cruzi*

**DOI:** 10.3389/fcimb.2022.901880

**Published:** 2022-06-30

**Authors:** Jessica Rodríguez-Durán, Juan Pablo Gallardo, Catalina Dirney Alba Soto, Karina Andrea Gómez, Mariana Potenza

**Affiliations:** ^1^ Laboratorio de Biología e Inmunología de las Infecciones por Tripanosomátidos, Instituto de Investigaciones en Ingeniería Genética y Biología Molecular “Dr. Héctor Torres”—CONICET, Buenos Aires, Argentina; ^2^ Instituto de Microbiología y Parasitología Médica, Departamento de Microbiología, Facultad de Medicina, Universidad de Buenos Aires, Buenos Aires, Argentina

**Keywords:** *Trypanosoma cruzi*, EF-hand, hypothetical protein, differentiation, host-cell invasion

## Abstract

In the pathogen *Typanosoma cruzi*, the calcium ion (Ca^2+^) regulates key processes for parasite survival. However, the mechanisms decoding Ca^2+^ signals are not fully identified or understood. Here, we investigate the role of a hypothetical Ca^2+^-binding protein named TcCAL1 in the *in vitro* life cycle of *T. cruzi*. Results showed that the overexpression of TcCAL1 fused to a 6X histidine tag (TcCAL1-6xHis) impaired the differentiation of epimastigotes into metacyclic trypomastigotes, significantly decreasing metacyclogenesis rates. When the virulence of transgenic metacyclic trypomastigotes was explored in mammalian cell invasion assays, we found that the percentage of infection was significantly higher in Vero cells incubated with TcCAL1-6xHis-overexpressing parasites than in controls, as well as the number of intracellular amastigotes. Additionally, the percentage of Vero cells with adhered metacyclic trypomastigotes significantly increased in samples incubated with TcCAL1-6xHis-overexpressing parasites compared with controls. In contrast, the differentiation rates from metacyclic trypomastigotes to axenic amastigotes or the epimastigote proliferation in the exponential phase of growth have not been affected by TcCAL1-6xHis overexpression. Based on our findings, we speculate that TcCAL1 exerts its function by sequestering intracellular Ca^2+^ by its EF-hand motifs (impairing metacyclogenesis) and/or due to an unknown activity which could be amplified by the ion binding (promoting cell invasion). This work underpins the importance of studying the kinetoplastid-specific proteins with unknown functions in pathogen parasites.

## Introduction

Chagas disease, or American Trypanosomasis, is caused by the parasite *Trypanosoma cruzi*, which is mainly transmitted by blood-sucking triatomine insects. Nearly seven million people are infected worldwide, mostly in Latin America, where this neglected disease is endemic but also in non-endemic countries due to migration movements (Lidani et al., 2019). Without treatment, Chagas disease progresses from an acute phase, characterized by high parasitemia, to chronicity. In the latter, patients can stay asymptomatic for the rest of their lives or, in ~40% of the cases, develop severe digestive, cardiac, and/or neurological pathology (World Health Organization, 2022). However, there is still no vaccine and the current chemotherapy has frequent toxic side effects, being poorly effective in the chronic phase (Hasslocher-Moreno et al., 2020). Therefore, safer and more effective drugs in the chronic phase would improve the outcome of the disease. Research on *T. cruzi* biology can identify novel metabolic targets in the parasite, leading to the development of new chemotherapeutic agents.

The *T. cruzi* life cycle involves the passage from an insect vector to a mammalian host and vice versa, where the parasite survives and proliferates by adapting to different environments. At least four main developmental forms can be morphologically identified in this journey. In the hindgut of the insect, proliferating non-infective epimastigote forms differentiate into infective cell cycle-arrested metacyclic trypomastigotes, a process called metacyclogenesis. These parasite forms are eliminated by the triatomine feces and deposited near the bite during the blood meal. The parasite entrance commonly occurs through a wound in the skin or mucosal membrane of the mammalian host. Once in the bloodstream, metacyclic forms invade different host cell types and tissues and then differentiate intracellularly into amastigotes, which proliferate by binary fission. When the host cell harbors a high number of parasites, amastigotes turn into highly motile trypomastigotes, which promote cell lysis and invade neighboring cells or reach the bloodstream to disseminate the infection. These bloodstream trypomastigotes can also be taken up by triatomine bugs when feeding on the blood of the mammalian host (Teixeira et al., 2012).

Ca^2+^ signaling modulates diverse biological activities in eukaryotic cells. Cytosolic Ca^2+^ binds to a plethora of effectors responsible for regulating different cell type-dependent processes, establishing unique signaling pathways for each cell (Berridge et al., 2003). Ca^2+^ binding proteins (CBP) are some of these effectors, exerting functions ranging from the maintenance of intracellular homeostasis to the modulation of enzymatic activity. Most CBPs are characterized by the presence of EF-hand domains, a highly conserved structure of helix-loop-helix responsible for binding Ca^2+^. Typically, these motifs occur in pairs, facilitating the cooperative binding of two ions per protein, although it is also possible to find CBP with an odd number of domains (Chazin, 2011).

Experimental evidence has pointed out the importance of Ca^2+^ signaling and homeostasis in regulating key processes of the *T. cruzi* life cycle, such as proliferation, differentiation, and parasite–host interplay (Yoshida, 2006; Caradonna and Burleigh, 2011; Hashimoto et al., 2016). It has been found that during metacyclogenesis, the cytosolic Ca^2+^ ([Ca^2+^]_c_) increases abruptly and temporarily regardless of the extracellular ion concentration, suggesting this signal is triggered by intracellular reservoirs (Lammel et al., 1996). Also, during the invasion of mammalian cells *in vitro*, measurements with fluorescent indicators showed that the [Ca^2+^]_c_ in trypomastigotes transiently increases as a consequence of parasite adhesion to the outer membrane of the host cell (Moreno et al., 1994; Yakubu et al., 1994). Furthermore, the [Ca^2+^]_c_ increase observed in tissue culture-derived trypomastigotes is also triggered by interaction with mammalian extracellular matrix proteins (Manchola Varón et al., 2021). Regarding this, the main intracellular reservoirs studied to date for Ca^2+^ storage and mobilization are the acidocalcisomes, the endoplasmic reticulum and the mitochondria (Lu et al., 1998; Maeda et al., 2012; Chiurillo et al., 2020). Nearly a dozen Ca^2+^ transport channels have been identified and/or characterized in these organelles, as well as in plasma or flagellar membranes (Furuya et al., 2001; Hamedi et al., 2015; Ramakrishnan and Docampo, 2018; Chiurillo et al., 2019; Dave et al., 2021), which handle the mobilization, flux, and extrusion of Ca^2+^ ions among the different compartments and the extracellular environment. The differences in structure, location, and regulation found in some of these channels in comparison to those present in mammalian cells, in addition to their relevance in parasite physiology, make these channels an attractive target for therapeutic treatment (Benaim et al., 2020). However, few CBP containing EF-hand domains have been characterized in *T. cruzi*. Among them, it has been shown that calmodulin modulates the activity of enzymes such as Ca^2+^ and Mg^2+^ dependent ATPases and cyclic AMP phosphodiesterase (Tellez-Iñón et al., 1985; Benaim et al., 1991). In addition, other CBPs have been characterized functionally to some extent. In this regard, the Ca^2+^/calmodulin-dependent kinase CaMK II, the calcineurin regulatory subunit B and the flagellar Ca^2+^-binding protein FCaBP have been implicated in epimastigote proliferation (Souza et al., 2009; Nogueira et al., 2011), mammalian host-cell invasion (Araya et al., 2008) and flagellum assembly (Wingard et al., 2008), respectively. Calreticulin, a CBP lacking EF-hand domains, was shown to play a role in *T. cruzi* infection (Sánchez-Valdez et al., 2014). In turn, Ca^2+^ modulates enzymatic activity in several proteins, such as the adenylyl cyclase, which is involved in parasite motility (D'Angelo et al., 2002). Overall, these studies demonstrated the presence of Ca^2+^ signaling in *T. cruzi* metabolism and physiology, although its actors and mechanisms are not fully identified or completely understood.

Since the annotation of *T. cruzi* genomes, several CBP and Ca^2+^ channels have been identified. These proteins probably generate the fluctuations in calcium concentration and decode its signals, although most of them remain uncharacterized (El-Sayed et al., 2005). Furthermore, many of these genes encode hypothetical proteins, which lack significant sequence homology in other eukaryotes. These trypanosomatid-exclusive novel proteins of unknown function represent ~39% of the protein-coding genes in the *T. cruzi* genome (Berná et al., 2018). Unraveling their role would lead to the identification of novel chemotherapeutic targets against these pathogens (Martins et al., 2011).

This study contributes to the knowledge of Ca^2+^ signaling in *T. cruzi*. For this purpose, we carried out a multi-step strategy search in the *T. cruzi* databases to identify the hypothetical Ca^2+^-binding proteins with experimental evidence of their expression. We selected one of these proteins, named TcCAL1, to investigate its role in the *T. cruzi* life cycle. To achieve this, parasites expressing different levels of TcCAL1 were evaluated regarding their proliferation, differentiation, and host cell invasion. Our results reinforce the importance of studying and revealing the function of hypothetical proteins, which may be relevant for parasite survival.

## Material and Methods

### Identification of Kinetoplastid-Specific Proteins and Phylogenetic Analysis

**Figure 1:**
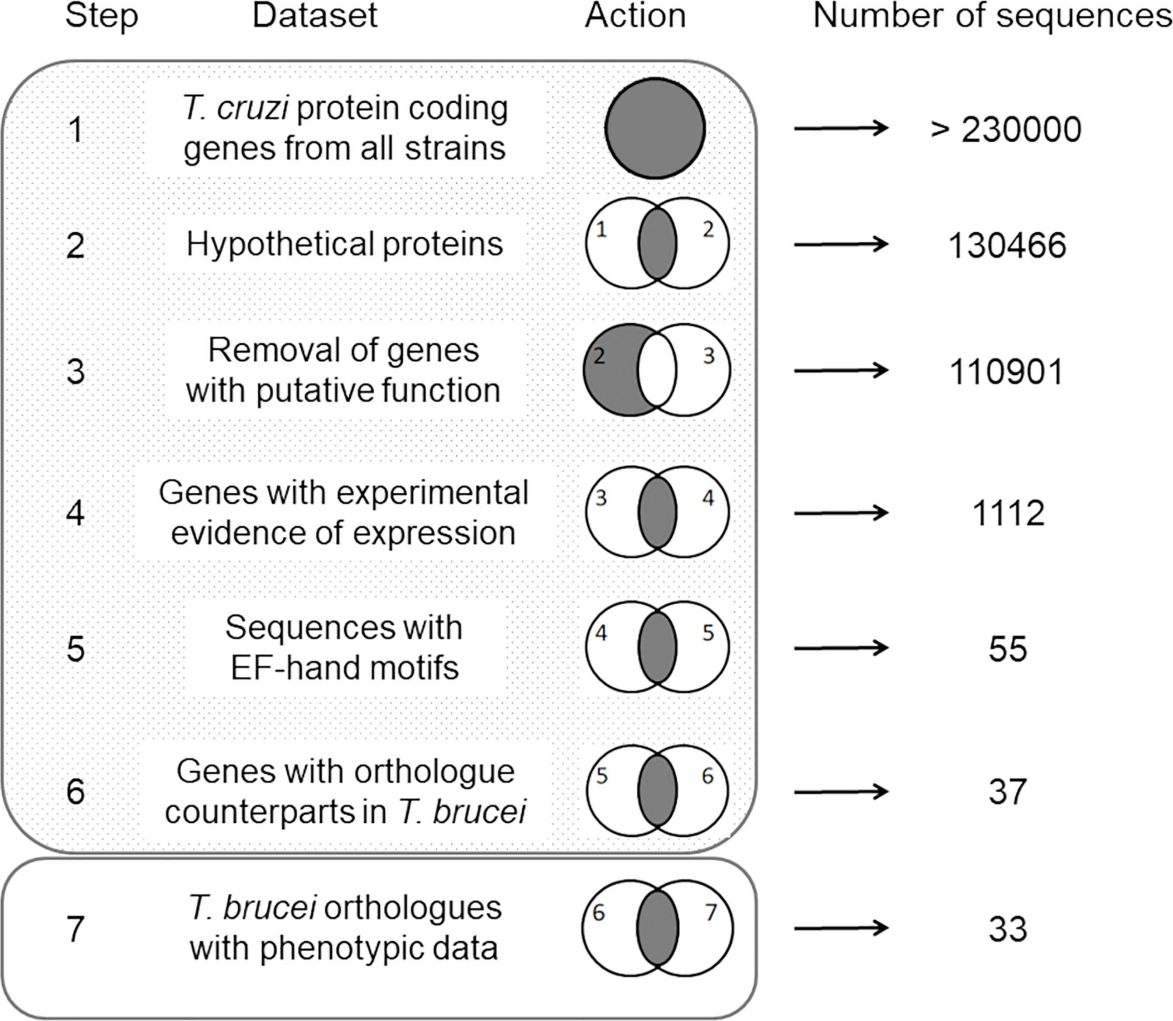
Schematic representation of the manual routine used in this study. The dataset used for sequence selection and the resulting number of genes are indicated for each step. Venn diagrams schematize the action performed. Shadowed and empty rectangles represent the TriTrypDB and TDRtargets databases, respectively, and indicate the website where the sequence searches were performed.

Searches for protein coding sequences were performed on the TriTrypDB and TDRtargets databases (Aslett et al., 2010; Alsford et al., 2011). Sequence processing was carried out following these user-defined steps: 1—selection of sequences annotated as hypothetical proteins in the *T. cruzi* genomes (last accessed March 1st, 2022), 2—removal of sequences with any putative function assigned, 3—selection of hypothetical proteins containing at least one EF-hand domain, 4—selection of proteins with experimental evidence of expression by proteome analysis, 5—selection of sequences having orthologues in *Trypanosoma brucei*, and 6—selection of sequences whose *T. brucei* orthologues revealed evidence of essentiality in parasite proliferation and differentiation. This strategy is schematized in **Figure 1**.

The evolutionary history of the TcCAL1 protein was constructed using the homologous protein sequences from parasites of the Kinetoplastid order retrieved from the TrytripDB database. The homologous sequence of *Bodo saltans* was used as the outgroup (Deschamps et al., 2011). Multiple sequence alignment of these sequences (MSA) was constructed using the MAFFT 7 server using BLOSUM 62 as the scoring matrix and visualized with the MEGA-X software (Katoh et al., 2019). The MSA was used to perform phylogenetic analysis using the Maximum Likelihood method in RAxML (Stamatakis, 2014). ProtTest 3.4 was used to calculate the phylogenetic parameters (Darriba et al., 2011). A 500-rapid bootstrap was selected to estimate the robustness of the phylogenetic inference. The transfer bootstrap expectations were calculated using BOOSTER (booster.pasteur.fr/) (Lemoine et al., 2018). FigTree (https://tree.bio.ed.ac.uk/software/figtree) was used to visualize and edit the trees. The protein structure prediction was performed using AlphaFold (Jumper et al., 2021) and visualized and edited using the molecular graphics system PyMOL (https://www.schrodinger.com/products/pymol). The ion binding sites were predicted using the COACH server (https://zhanggroup.org/COACH/).

### Recombinant Protein Expression and Polyclonal Antiserum Production

The coding sequence of TcCAL1 was amplified from *T. cruzi* genomic DNA by PCR using the primers 5´-GCGCCATGGCTATGCAACGCAGTCTAAT-3 and 5´-GCGGTCGACTCGCTG AGCAAAATT-3´. The amplified fragment was cloned into the *NcoI-SalI* restriction sites of the pET22 expression vector, fused to a C-terminal six histidine tag (6xHis), and transformed into BL21 pLysS *E. coli* cells. The recombinant protein TcCAL1-6xHis was purified from bacterial extracts by affinity chromatography using a nickel-charged agarose resin (Ni-NTA Agarose, Qiagen). Four adult male BALB/c mice were immunized with three boosts of 30–50 µg of TcCAL1-6xHis each. A pre-immune sample was taken from the mice prior to immunization,. Fourteen days after the last boost, the mice were bled and the antisera were obtained. Procedures concerning animal treatments in this study were carried out in accordance with the bioethics guidelines of the National Research Council regarding the care and use of laboratory animals (Garber, 2011).

### Parasite Culture and Samples

*T. cruzi* epimastigote forms from the Discrete Typing Units DTUI and DTUII (hereafter named CL and Y, respectively), were grown at 28°C in Liver Infusion Tryptose medium (LIT) supplemented with 10% Fetal Bovine Serum (FBS) (Natocor), 20 mM glucose, 20 µM hemin (Sigma-Aldrich), 10 U/ml Penicilin and 10 mg/ml Streptomycin (*In vivo*gen). Samples of epimastigotes, cell culture-derived trypomastigotes, and Vero cells infected with intracellular amastigotes from the *T. cruzi* Brazil strain were kindly provided by Dr. Cecilia Albareda.

### Overexpression of TcCAL1-6xHis in *T. cruzi*

To generate transgenic parasites, the sequence coding for TcCAL1-6xHis was isolated by PCR from the pET22 recombinant vector generated in *Recombinant Protein Expression and Polyclonal Antiserum Production* using the primers 5´-GCTCTAGAATGCAACGAGTCTAAT-3´ and 5´-GCGCATCGATCTCAGTGGTGGTGGT-3´, and sub-cloned into the *XbaI-XhoI* restriction sites of the pTREX expression vector (Vazquez and Levin, 1999). The recombinant plasmid pTREX/TcCAL1-6xHis was amplified in DH5α *E. coli* cells and purified using the PureYield™ Plasmid Midiprep System (Promega, USA). *T. cruzi* epimastigotes from CL or Y strains were transfected with pTREX/TcCAL1-6xHis following a standard electroporation protocol (Potenza et al., 2012). Early mid-log phase epimastigote cultures were washed twice with cold phosphate saline buffer (PBS; 39 mM Na_2_HPO_4_, 10 mM NaH_2_PO_4_, 137 mM NaCl, 22 mM KCl, pH 7.4) and resuspended in 350 µl of cold transfection buffer (0.1 Mm of CaCl_2_, 0.5 of mM MgCl_2_, and 272 of mM sucrose in PBS, pH 7.2) at a density of 1 × 10^8^ parasites/ml. The cells were mixed with 40 μg of DNA, transferred to 0.2 mm electroporation cuvettes, and incubated on ice for 5 min. Parasites were electroporated with one pulse at 400 V and 500 μF using a Gene Pulser^®^ II electroporator (Biorad, USA), recovered on ice 5 min and then incubated in 7 ml of LIT supplemented with 20% of FBS at 28°C overnight. The selection of recombinant parasites was performed for 4 weeks in the presence of increasing concentrations of Geneticin (G418, InvivoGen) up to 500 μg/μl. To perform the culture controls, the transfection and selection procedures were performed using the empty vector pTREX on epimastigotes from CL or Y strains.

### Proliferation Assays

A total of 1 × 10^5^ epimastigotes/ml from CL or Y *T. cruzi* cultures stably transfected with TcCAL1-6xHis or the empty vector pTREX (hereafter named CL-CAL1 and CL-C or Y-CAL1 and Y-C, respectively) were grown in LIT supplemented with G418. Cultures were incubated at 28°C over time and samples were taken at 24 or 48 h intervals. The number of parasites was counted in triplicate using a Neubauer chamber and an optical microscope. Growth curves were expressed in the number of parasites per ml.

### Metacyclogenesis Assays

Epimastigote forms from CL-C and CL-CAL1 or Y-C and Y-CAL1 cultures were differentiated into metacyclic trypomastigotes using the protocol described by Rodríguez Durán et al. (2021). Briefly, ~3 × 10^7^ epimastigotes growing in 3 ml of LIT supplemented with G418 were added to 15 ml-conical tubes containing blood-agar slants (generating a biphasic medium), and incubated at 28°C for three weeks. Parasites transformed into metacyclic trypomastigotes as well as non-differentiated epimastigotes were counted in triplicate for each culture. Epimastigotes from the Y strain were also differentiated using the Triatomine Artificial Urine (TAU) method (Contreras et al., 1985). For this, a 7-day-old epimastigote culture was incubated in TAU medium (190 mM of NaCl, 17 mM of KCl, 2 mM of MgCl_2_, 2 mM of CaCl_2_, and 8 mM of sodium phosphate buffer, pH 6.0, all from Biopack) for 2 h at room temperature. Then, the parasites were pelleted by centrifugation, resuspended in TAU-3AAG medium (TAU supplemented with 50 mM sodium glutamate, 10 mM L-proline, 2 mM sodium aspartate, and 10 mM glucose, all from Sigma-Aldrich) and incubated for up to 72 h at 28°C. Every 24 h, metacyclic forms were counted in triplicate. For both protocols, the percentage of metacyclogenesis was calculated using the formula: (metacyclic trypomastigote number × 100)/(metacyclic trypomastigote number + epimastigote number).

### Metacyclic Trypomastigotes Isolation

Y-C or Y-CAL1 metacyclic trypomastigotes from the biphasic medium were isolated from the remaining non-differentiated epimastigote forms by complement-mediated parasite lysis, as previously described (Cestari et al., 2009). In brief, the liquid phase of the biphasic medium containing both the metacyclics and the non-differentiated epimastigote forms was centrifuged and resuspended in 800 µl of non-heat inactivated FBS diluted in 200 µl of RPMI medium. After incubation at 37°C for 1 h, the suspension was centrifuged again to pellet the metacyclic forms and the cellular debris generated by the epimastigote lysis. Then, the sample was resuspended in RPMI supplemented with 5% heat-inactivated FBS, incubated for 3 h at 37°C to allow the motile metacyclic trypomastigotes to swim back to the supernatant, and finally transferred to a new tube. Purified metacyclic trypomastigotes were washed thoroughly with RPMI supplemented with FBS prior to downstream applications. Centrifugations were performed at 3,000**×** *g* for 10 min.

### Adhesion and Invasion Assays

Metacyclic trypomastigotes isolated as described in *Metacyclic Trypomastigotes Isolation* were used to infect Vero cells at a 300:1 parasite:host cell ratio. Due to the differences in multiplicity of infection among Y-C and Y-CAL1 parasites, the 300:1 ratio allowed us the comparison of the following parameters. For adhesion assays, 6 × 10^6^ parasites were placed on 13 mm round glass coverslips coated with 2 × 10^4^ Vero cells and incubated for 2 h at 37°C followed by five washings. Coverslips were stained with ColorPack panoptic dye kit (BioPack) following the instructions of the supplier. Stained cells were visualized and photographed using an optical microscope to calculate the percentages of cells containing adhered parasites and the number of parasites per host cell in randomly selected fields. A total of 200 host cells were counted in triplicate. For invasion assays, a similar experimental procedure was carried out by incubating parasites with Vero cells for 24 h. After 48 h post-infection, coverslips were stained with Giemsa and observed using a microscope to calculate the percentages of infected cells and the number of intracellular amastigotes per host cell in randomly selected fields. Here, 300 host cells were counted in triplicate. All incubations were made in RPMI plus 5% FBS. All washes were performed using PBS. The adhesion index was calculated by multiplying the percentage of Vero cells with attached parasites by the mean number of adhered parasites per cell. The invasion index was determined by multiplying the percentage of infected Vero cells by the mean number of amastigotes per infected cell. The index-fold change (FC) was calculated using the formula FC = (I_Y-CAL1_ − I_Y-C_)/I_Y-C_, where I_Y-CAL1_ or I_Y-C_ refer to the adhesion or invasion indeces for Vero cells incubated with Y-C or Y-CAL1 parasites, respectively.

### Amastigogenesis Assay

*In vitro* amastigogenesis was performed following the protocol described elsewhere (Hernández-Osorio et al., 2010). For example, 1 × 10^7^ metacyclic trypomastigotes were collected by centrifugation at 3,000**×** *g* for 10 min. Pellets were subsequently washed three times with MEM pH 7.4 and twice with MEM pH 5, in the absence of FBS. Parasites were then resuspended in 100 µl of MEM pH 5 supplemented with 0.4% BSA and incubated at 37°C. Samples were taken at 0, 4, and 6 h and processed as described in *Immunofluorescence Microscopy* for microscopic analysis. The percentages of axenic amastigotes and intermediate or non-differentiated forms were determined by counting 300 parasites in randomly selected fields per sample.

### Immunofluorescence Microscopy

*T. cruzi*-infected Vero cells grown on round glass coverslips or parasites adhered to poly-lysine coated microscope slides were fixed with 4% paraformaldehyde for 30 min, permeabilized with 0.1% Triton X-100 for 3 min, and washed twice. After blocking samples in 2% bovine serum albumin (BSA), slides were incubated with the anti-TcCAL1 or pre-immune antiserum, an anti-paraflagellar rod mouse monoclonal antibody (anti-PFR, kindly provided by Dr. S. Schenkman) to reveal the flagellum or an anti-tubulin rat antibody, clone YL1/2 (anti-TUB, Chemicon) to detect the parasite body by its affinity to tyrosinated tubulin (diluted 1:300, 1:2, or 1:500, respectively, in 1% BSA). The secondary antibodies were Alexa Fluor 488-conjugated goat anti-mouse (Invitrogen) or Cy3-conjugated goat anti-rat (Jackson), both diluted 1:1,000. All washes and compound dilutions were performed in PBS. All incubations lasted for 1 h each at room temperature. Slides were mounted on Vectashield^®^ Antifade reagent (Vector Laboratories), containing 10 mg/ml of 4,6-diamino-2-phenylindole (DAPI). The Cells were observed using an Olympus BX-61 fluorescence microscope.

### Protein Extracts and Western Blot

To obtain protein parasite extracts, epimastigotes or metacyclic forms were harvested by centrifugation at 1,000**×** *g*, washed with PBS, resuspended in cold cell lysis buffer containing 50 mM Tris–HCl pH 7.5, 14 mM β-mercaptoethanol, protease inhibitor cocktail (Roche), and disrupted by six freezing-thawing cycles in liquid nitrogen. Protein-soluble fractions were separated from the cellular debris by centrifugation at 12,000**×** *g* for 10 min at 4°C and then quantified using Bradford’s method (Bradford, 1976). Pellets containing cellular debris were solubilized by the addition of 5× SDS-PAGE sample buffer (0.25 M Tris–HCl pH 6.8, 0.5 M DTT, 10% SDS, 50% Glycerol, 0.5% bromphenol blue) up to an equal volume of soluble fraction. All samples were separated on 15% SDS-PAGE polyacrylamide gels and then transferred to nitrocellulose membranes (Amersham Biosciences). The membranes were blocked with 5% non-fat milk in PBS for 1 h and then incubated overnight at 4°C with anti-TcCAL1 or mouse monoclonal anti-α-tubulin (Sigma Aldrich) diluted 1:300 or 1:8,000, respectively. The membranes were then washed four times with PBS-0.1% Tween 20 and incubated with peroxidase-conjugated goat anti-mouse IgG secondary antibody (Kirkegaard & Perry Laboratories KPL), diluted 1:1,000 for 1 h. Incubations were performed at room temperature unless otherwise indicated. Protein bands were revealed using Western Lightning^®^ Plus-ECL (Perkin Elmer) chemiluminescence reagent and visualized on a GeneGenome XRQ device (Syngene).

### Statistics

All statistical analysis was performed using the tests indicated in figure legends and the GraphPad Prism 5.0 (GraphPad Software). All data were considered statistically significant at p <0.05 and values are expressed as means ± standard deviation (SD).

## Results

### TcCAL1 is a Kinetoplastid-Specific Protein With Predicted Ca^2+^ Binding

**Figure 2:**
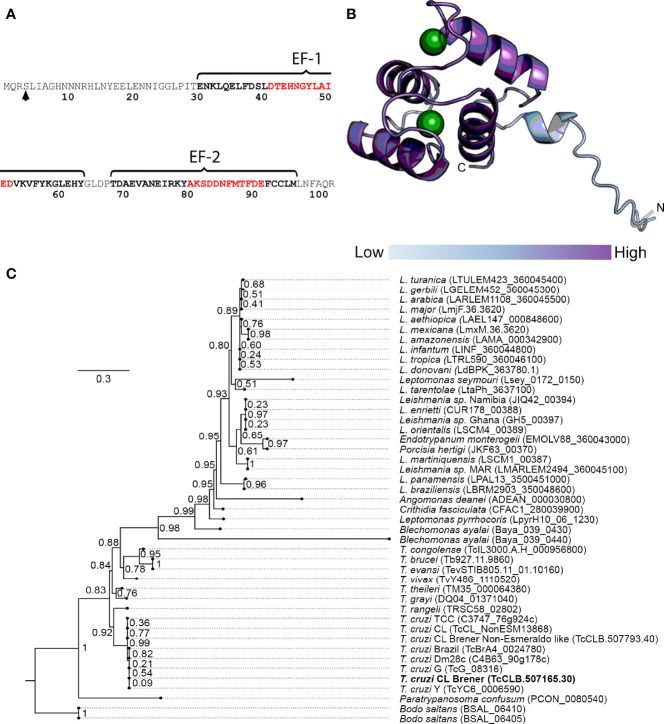
Sequence features of TcCAL1 and a phylogenetic tree. **(A)** Amino acid sequence and position of the EF-hand motifs. Residues in black and red bold indicate the alpha helices and the Ca^2+^ binding cleft, respectively. The arrow indicates the phosphorylated serine. **(B)** Predicted tridimensional structure of TcCAL1. The circles represent Ca^2+^ ions. The different color intensity indicates the model confidence, being high at the C-terminus and lower at the N-terminus (dark and light blue, respectively). **(C)** TcCAL1 evolutionary relationship with different species of trypanosomatids. The scale-bar represents 0.3 substitutions per position in the aminoacid sequence. The numbers at the branches indicate bootstrap values. TcCAL1 is highlighted in bold.

To address the identification of novel proteins potentially involved in *T. cruzi* Ca^2+^ signaling, we performed user-defined sequence searches at the TriTrypDB web site, a database containing the annotation of several kinetoplastid genomes integrated into bioinformatics resources and experimental datasets. Therefore, we identified gene sequences from *T. cruzi* that codify for proteins containing at least one EF-domain for Ca^2+^ binding and exhibited experimental evidence of their expression by proteome analysis. The proteins with any putative function were excluded from the dataset by removing the coding sequences containing the term “*putative*” in their annotation. Based on data from RNAi experiments (Alsford et al., 2011), we selected the *T. cruzi* proteins that had orthologue counterparts and experimental evidence of essentiality in *T. brucei*. As a result, 33 proteins were identified and one of them was selected for further study (TcCLB.507165.30). This protein, named TcCAL1, is 103 amino acid-long, with two predicted EF-hand motifs spanning the residues 31–97 and one phosphorylation site at serine 4 (Marchini et al., 2011) (**Figure 2A**). *Ab initio* protein structure prediction using AlphaFold allowed us to assign the putative tridimensional folding only in the EF-hand regions of the protein, since the first 31 amino acids did not match to any other structure with confidence enough to make a prediction (**Figure 2B**). Other TcCAL1-orthologues were identified in other *Trypanosoma* species and *Leishmania* genera, as well as in the free-living *B. saltans*. The phylogenetic relationships inferred from the tree based on TcCAL1-orthologues fit well with the current taxonomy of kinetoplastids (**Figure 2C**). However, in BLAST searches at NCBI, no clear orthologues were found in organisms other than kinetoplastids.

### TcCAL1 Localization in *T. cruzi*

**Figure 3:**
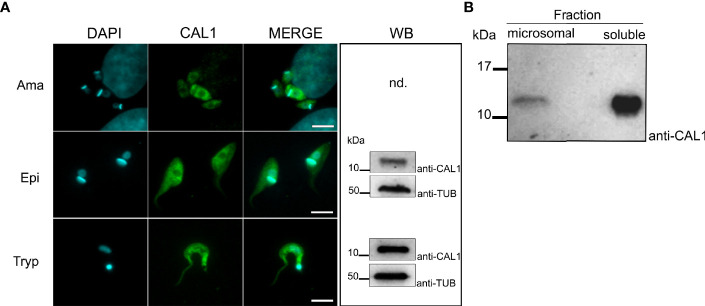
Expression and localization of endogenous TcCAL1. **(A)** Immunofluorescence microscopy of different stages of *T. cruzi* from the Brazil strain. Ama, Epi, and Tryp denote amastigote, epimastigote, and trypomastigote forms, respectively. DAPI and anti-CAL1 indicate staining of the nuclei and kinetoplast, and the signal corresponding to the anti-TcCAL1-6xHis serum, respectively. Scale bar: 7 µm. WB, Western blot analysis of whole-cell extract from the corresponding developmental form using the anti-TcCAL1-6xHis serum. Nd, not determined (due to limited sample size). **(B)** WB analysis of microsomal and soluble fractions from epimastigote protein lysates. In panels **(A, B)**, 30 µg of total protein extract was loaded in each lane. Nitrocellulose membranes were incubated with the anti-TcCAL1-6xHis antiserum (anti-CAL1). In **(A)**, an anti-Tubulin antibody (Anti-TUB) was used as a loading control. Band signals appeared at the expected sizes. kDa, kiloDaltons.

To reveal the subcellular localization of TcCAL1 in the main *in vitro* developmental forms of *T. cruzi*, we used a polyclonal antiserum against the purified recombinant TcCAL1-6xHis produced in *E. coli* by immunofluorescence microscopy. Images showed that TcCAL1 was present along the entire body of the parasite cell but to a lesser extent in organelles containing the nuclear and mitochondrial DNA (**Figure 3A**). In accordance, western blot (WT) of protein extracts using the anti-TcCAL1-6xHis antibody also revealed that the signal corresponding to TcCAL1 was mainly present in the soluble fraction of epimastigote forms (**Figure 3B**).

### Effect of TcCAL1-6xHis Overexpression on *T. cruzi* Proliferation and Differentiation

**Figure 4:**
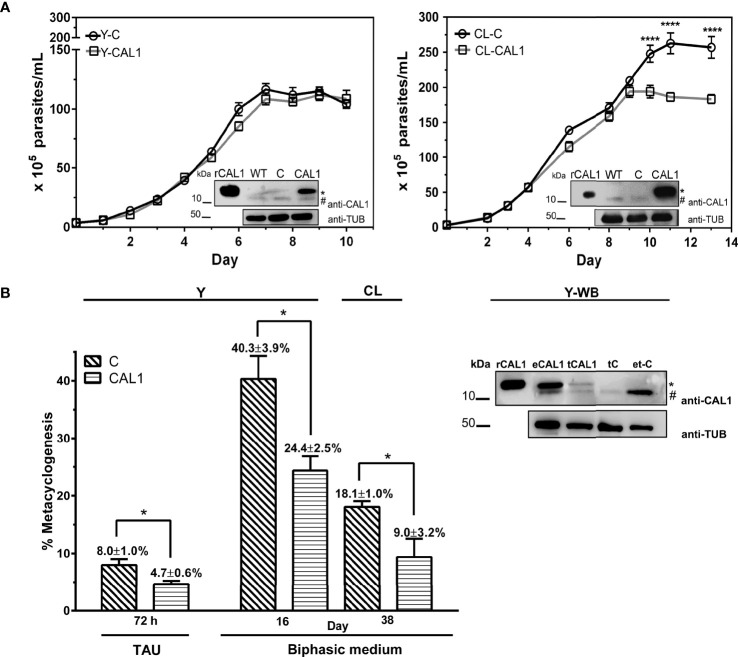
Effect of TcCAL1-6xHis overexpression on epimastigote proliferation and differentiation. **(A)** Growth curves of TcCAL1-6xHis overexpressing parasites from Y and CL strains. Epimastigote cultures overexpressing TcCAL1-6xHis (CAL1) or transfected with the pTREX empty vector (C) were counted over time. Statistical analyses were performed using a one-way ANOVA test. ****p <0.0001. At the inset of each curve, WB shows the overexpression of TcCAL1-6xHis in *E coli.* and WT, C rCAL1, recombinant CAL1 and CAL1, protein extracts from wild type, controls, and TcCAL1-6xHis-overexpressing parasites, respectively. A total of 20 µg of total protein extracts were loaded into each well. **(B)** Metacyclogenesis rates for parasites from Y and CL strains overexpressing TcCAL-6xHis and the respective controls. The method used to differentiate epimastigotes into metacyclic trypomastigotes is indicated (TAU or biphasic medium). Statistical analyses were performed using the Student’s t-test. *p <0.05. In the right panel, WB analysis of Y strain shows the expression of TcCAL1-6xHis in metacyclic trypomastigotes from biphasic medium. eCAL1 and tCAL1, epimastigotes and metacyclic trypomastigotes expressing TcCAL1-6xHis, respectively; tC, control metacyclics carrying the empty vector pTREX; e-tC, isolation sample control (prepared with trypomastigotes transfected with pTREX after incubation and subsequent washes of TcCAL1-6xHis-overexpressing epimastigote protein lysates). A total of 15 µg of total protein extracts were loaded into each well. In panels **(A, B)**, graphs and WB show one representative result of four and three independent experiments, respectively. The anti-TcCAL1-6xHis antiserum (anti-CAL1) was used to detect the recombinant protein and the endogenous TcCAL1 (indicated with a dot and a numeral, respectively). An anti-Tubulin antibody (Anti-TUB) was used as a loading control. kDa, kiloDaltons.

To address the functional characterization of TcCAL1, we first analyzed the effect of its over-expression on the proliferation and differentiation of *T. cruzi* epimastigotes. For this, we generated transgenic cultures overexpressing the fusion protein TcCAL1-6xHis or harboring the empty vector pTREX, used as a control. The effect of TcCAL1-6xHis overexpression on proliferation was determined by measuring the number of epimastigotes per ml of culture over time. Growth curves showed no significant differences in proliferation during the exponential phase in TcCAL1-6xHis overexpressing parasites compared to controls for both Y and CL strains (**Figure 4A**). However, significant differences were found in the stationary phase of growth in the CL strain. TcCAL1-6xHis overexpressing parasites stopped growing on day 9 of culture, while control parasites continued to divide. This pattern was observed until day 11, when the number of parasites in TcCAL1-6xHis-overexpressing and control cultures was 1.75 × 10^7^ *versus* 2.5 × 10^7^ epimastigotes/ml, respectively.

Then, we moved forward to study the effect of TcCAL1-6xHis overexpression on the metacyclogenesis process by performing differentiation assays in biphasic medium. The metacyclogenesis rates for parasites overexpressing TcCAL1-6xHis and controls were calculated after 19 days, when the number of metacyclics in both cultures had risen to the maximum. The better performance in differentiation for the CL strain was found on day 38. Therefore, metacyclogenesis rates were calculated after this period of incubation (**Figure 4B**). Results showed a significant decrease in metacyclogenesis percentages in TcCAL1-6xHis overexpressing parasites compared to controls for both strains (Y: 24.4 ± 2.5 vs 40.3 ± 3.9; CL: 9.0 ± 3.2 vs 18.1 ± 1.0). In parallel, we also performed differentiation assays for the Y strain following the TAU protocol. Although the total number of metacyclics reached by this method was lower than that of the biphasic medium, we found that the metacyclogenesis was also impaired in TcCAL1-6xHis overexpressing parasites compared to controls (4.7 ± 0.6 vs 8.0 ± 1.0). WB analysis corroborated the expression of TcCAL1-6xHis in metacyclics obtained after differentiation in biphasic medium for the Y strain (tCAL1, **Figure 4B**). Here, a sample control (e-tC, **Figure 4B**) was performed to confirm that the signal detected in WB corresponding to either the endogenous TcCAL1 or the recombinant TcCAL1-6xHis came from metacyclic trypomastigotes and not from residual complement-lysed epimastigote debris. For this, epimastigotes overexpressing TcCAL1-6xHis were mixed with metacyclic trypomastigotes carrying the empty pTREX vector (controls) and complement-mediated lysed by the addition of non-heat inactivated FBS. Then, the sample was washed to remove the TcCAL1-6xHis from epimastigote forms and subjected to WB analysis. The absence of signal at the TcCAL1-6xHis expected size indicated that the proteins detected in all samples were derived exclusively from metacyclic trypomastigotes.

**Figure 5:**
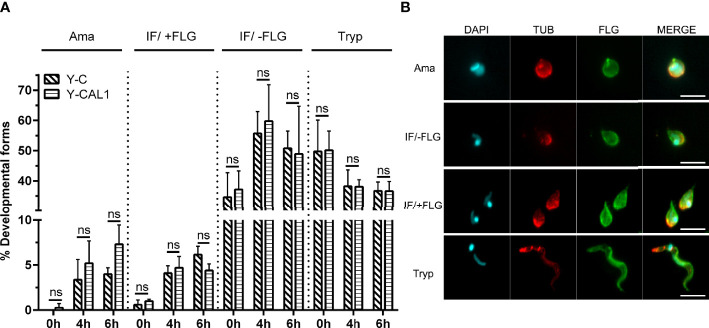
Effect of TcCAL1-6xHis overexpression on *in vitro* amastigogenesis. **(A)** Metacyclic trypomastigotes from Y strain expressing TcCAL1-6xHis or carrying the empty vector (Y-CAL1 or Y-C, respectively) were incubated in low pH medium. Samples were taken at 0, 4, and 6 h for counting and examination of the different morphological forms. The graph shows the percentage of each parasite form at different times. Ama, Tryp, IF/+FLG and IF/-FLG indicate axenic amastigotes, metacyclic trypomastigotes, and intermediate forms with or without flagellum, respectively. Statistical analyses were performed using Student’s t-test. ns, no significant. This graph shows one representative result of two independent experiments. **(B)** Immunofluorescence microscopy showing the morphology of each developmental form. DAPI staining reveals the kinetoplastid and nuclear DNA. TUB and FLG indicate incubation with anti-Tubulin or anti-PFR antibody, respectively.

We further wondered whether TcCAL1-6xHis overexpression would alter the differentiation of metacyclic trypomastigotes into axenic amastigotes. Hence, we performed amastigogenesis assays in which we incubated parasites from the Y strain in a low pH medium without serum (**Figure 5A**). Samples were taken at different times and, to better visualize the parasite morphology, immunofluorescence microscopy was carried out to reveal the nuclear and kinetoplastid DNA, as well as the presence or absence of the flagellum. Besides trypomastigotes and amastigotes, we also counted the intermediate forms, which are rounded parasites with a relative position of nuclear and kinetoplast DNA similar to trypomastigotes with or without a flagellum (IF/+FLG or IF/-FLG, respectively) (**Figure 5B**). Although the number of amastigotes increased over time, no significant differences were found in amastigogenesis rates for TcCAL1-6xHis overexpressing parasites compared with controls in the period analyzed.

### Overexpression of TcCAL1-6xHis Enhances the Infectivity of *T. cruzi*

**Figure 6:**
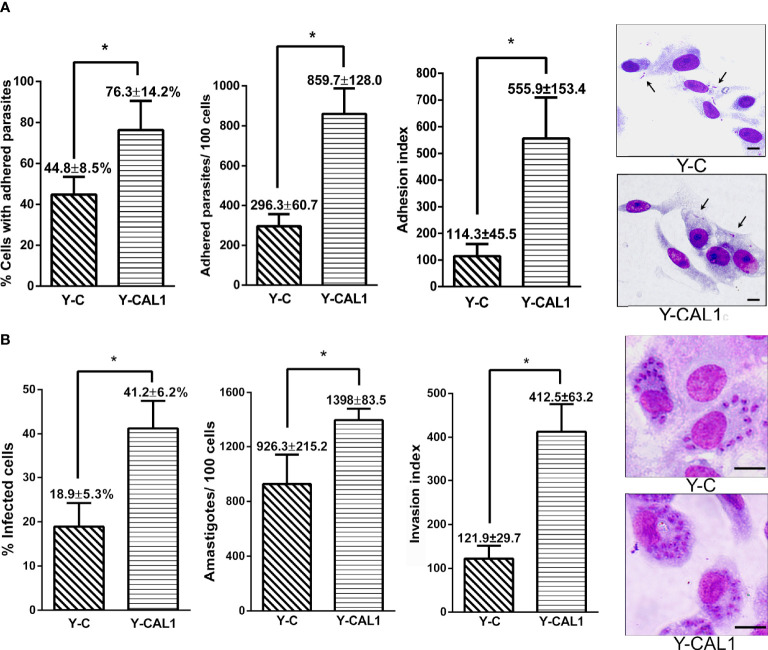
Effect of TcCAL1-6xHis expression on the infectivity of *T. cruzi*. **(A)** *In vitro* adhesion assay. Metacyclic trypomastigotes from Y strain expressing TcCAL1-6xHis or carrying the empty vector (Y-CAL1 or Y-C, respectively) were incubated with Vero cells for 2 h to calculate the percentage of cells with adhered parasites, the number of parasites adhered per 100 cells, and the adhesion index. On the right panel, panoptic stained cultures for each condition. Black arrows indicate some adhered parasites. **(B)** *In vitro* invasion assays. Metacyclic trypomastigotes from Y strain expressing TcCAL1-6xHis or carrying the empty vector (Y-CAL1 or Y-C, respectively) were incubated with Vero cells to calculate the infection percentages, determined by counting the number of infected cells after 48 h post-infection. The total number of internalized amastigotes in 100 Vero cells, as well as the invasion indexes, are also shown. Onthe right panel, Giemsa staining of infected Vero cells. In panels **(A, B)**, statistical analyses were performed using Student’s t-test. ^*^p <0.05. Data from one representative experiment of three independent experiments is shown. Scale bar, 10 µm.

We next evaluated the effect of TcCAL1 on the interaction with the mammalian host *in vitro* by adhesion assays. To that end, TcCAL1-6xHis-expressing metacyclic trypomastigotes from the Y strain, obtained by differentiation in biphasic medium, were incubated with Vero cells for 2 h, washed, and then stained for analysis and counting. The results showed that the percentage of cells with adhered parasites was significantly higher in mammalian cultures incubated with transgenic trypomastigotes than in controls (76.3 ± 14 vs 44.8 ± 3.5, respectively). Consistently, the number of parasites adhered per Vero cell was significantly higher for cultures incubated with trypomastigotes expressing TcCAL1-6xHis compared to controls (8,603 ± 128 vs 296 ± 60, respectively) (**Figure 6A**). We then assessed the effect of TcCAL1-6xHis overexpression during the host cell invasion process. Thus, isolated metacyclic trypomastigotes overexpressing TcCAL1-6xHis or controls were loaded on coverslips coated with Vero cells for 24 h following subsequent washes. After 48 h post infection, cultured cells were analyzed to compare the invasion capacity of parasites. As shown in **Figure 6B**, the percentages of infected cells, as well as the number of internalized amastigotes, were significantly higher in Vero cells infected with TcCAL1-6xHis-expressing trypomastigotes than in controls (41.2 ± 6.23 vs 18.9 ± 5.34% and 1,398 ± 48 vs 926 ± 124 parasites/100 host cells, respectively).

Overall, both the adhesion and invasion processes were promoted in Vero cultures incubated with TcCAL1-6xHis overexpressing parasites compared to controls. Nevertheless, the fold change comparing transgenic and control cultures was 3.96 for the adhesion index, whereas for the invasion index it was 2.21. This finding suggests a more pronounced beneficial effect of recombinant TcCAL1-6xHis during the attachment of parasites to the surface of Vero cells compared to the internalization process.

## Discussion

Herein, we explored the role of TcCAL1, a novel protein which renders both evidence of expression and putative Ca^2+^ binding domains, throughout the *in vitro* life cycle of *T. cruzi*. Our data showed that the overexpression of a tagged version of TcCAL1 impaired the metacyclogenesis of epimastigotes and enhanced the infectivity of metacyclic tripomastigotes to invade Vero cells. However, this overexpression had no substantial effects on the exponential proliferation of epimastigote forms or on the differentiation of metacyclic trypomastigotes into amastigotes. These findings reveal that TcCAL1 protein levels may exert a relevant role in *T. cruzi* metacyclogenesis and host-cell invasion.

The number of peptides found in datasets from proteomic studies (Atwood et al., 2005; Marchini et al., 2011; Queiroz et al., 2014; Jesus et al., 2017), and the presence of the endogenous TcCAL1 on our WB (**Figure 3B**), show evidence of its abundance in this organism. It could also be possible that TcCAL1 may not be tightly regulated during parasite proliferation. Even more, TcCAL1-6xHis overexpression was non-detrimental for other organisms since we were able to produce this recombinant protein in *E. coli* and *Leishmania*. That said, the curve for the CL strain exhibited a faint but significant phenotypic difference after the exponential growth, in which parasites overexpressing TcCAL1-6xHis reached the stationary phase earlier in comparison to control cultures. Previously, it was shown that under starving conditions, epimastigote proliferation becomes more sensitive to Ca^2+^ availability for maintaining mitochondrial bioenergetics (Chiurillo et al., 2019). Here, independent or synergistic mechanisms would explain this slightly lower performance in the cell density achieved by TcCAL1-6xHis-overexpressing cultures. Among them, a reduced cytosolic Ca^2+^ availability due to ion-sequestering by TcCAL1-6xHis would lead to early parasite starvation. A dominant negative effect exerted by the tagged version of TcCAL1, which is evidenced only at high levels of expression and culture densities, would also impair the parasite growth. By contrast, no difference was observed at the stationary stage of growth between control and TcCAL1-6xHis-overexpressing epimastigotes from Y strain. In comparison to CL, parasites from Y strain rendered lower culture densities (~2.7 × 10^7^ vs 1.2 × 10^7^, respectively) and recombinant TcCAL1-6xHis expression levels (see insets in **Figure 4A**). Both aforesaid evidences would explain the absence of measurable changes in the growth curves of transgenic and control parasites from Y strain, in addition to the intrinsic differences that cultures belonging to different DTU have in response to the same stimulus or condition (Ruiz et al., 1998; Majeau et al., 2021).

Along with a change in the morphology and the metabolic state, an abrupt but transitional increase in the cytosolic Ca^2+^ occurs during *T. cruzi* metacyclogenesis (Lammel et al., 1996; Hashimoto et al., 2016; Gonçalves et al., 2018). Several Ca^2+^- or Ca^2+^-activated channels involved in metacyclogenesis and other processes have been identified in the parasite (Furuya et al., 2001; Ramakrishnan and Docampo, 2018; Chiurillo et al., 2019; Dave et al., 2021). Meanwhile, few Ca^2+^-binding or -regulated proteins have been characterized so far (Belaunzarán et al., 2009; Hamedi et al., 2015; Lander et al., 2018), and three proteins involved in calcium homeostasis have been shown to play a role in metacyclogenesis: MICU1 and MICU2 (Bertolini et al., 2019) and Letm1 (Dos Santos et al., 2021). Here, we found that the metacyclogenesis rates were significantly lower in parasites overexpressing TcCAL1-6xHis for both CL and Y strains. One possible explanation may rely on an exacerbated Ca^2+^ chelating-like effect exerted by TcCAL1-6xHis, which in turn limits the availability of this ion in the cytosol. As a result, this limiting cytosolic Ca^2+^ level would downregulate the function of key enzymatic activities needed for parasite differentiation. On the other hand, TcCAL1 is a relatively small protein containing two EF-hand motifs located at 31–97 amino acid positions. There is no other putative functional domain in its sequence, and the AlphaFold algorithm failed to predict the tridimensional structure in the region spanning the amino acids 1 to 30, probably due to a high sequence divergence. EF-hand-containing proteins are a huge family of sequences with a broad range of functions (Chazin, 2011). In nature, small proteins containing one pair of EF-hands can act not only as Ca^2+^ buffering systems, but also exhibit catalytic functions or binding proprieties, as in the case of calmoduline, S100 proteins, or parvalbumin (Haiech et al., 2019; Mohanta et al., 2019; Elíes et al., 2020). If TcCAL1 has a catalytic or functional domain besides the EF-hand motifs, which in turn could be modulated upon Ca^2+^ binding, relevant for metacyclogenesis, is an important issue that needs further investigation. Certainly, the determination of Ca^2+^-binding properties of TcCAL1 by circular dichroism, the measurement of Ca2+-binding association/dissociation constants, and the analysis of conformational changes upon Ca^2+^ binding by magnetic resonance would address some of the hypotheses raised here (Pinto et al., 2003; Shan et al., 2018). It is worth mentioning that the effect of TcCAL1-6xHis overexpression on metacyclogenesis has been observed in both strains analyzed using two different methods. In fact, the percentages of metacyclic trypomastigotes overexpressing TcCAL1-6xHis from CL or Y strains significantly decreased ~50 and 40%, respectively, compared to control cultures. In fact, Y cultures followed the same behavior when incubated in TAU medium, resulting in ~40% fewer metacyclics in TcCAL1-6xHis overexpressing parasites than controls. Unfortunately, it was not possible to differentiate CL parasites using the TAU medium, probably because years of culturing as epimastigote forms dampened their response to external stimulus.

The effect of TcCAL1-6xHis on the *in vitro T. cruzi* life cycle was further investigated in host-cell invasion assays. We showed that the overexpression of TcCAL1-6xHis enhanced metacyclic adhesion to Vero cells as well as the infectivity of *T. cruzi*. At this point, it is important to mention that we chose the hexa-histidine sequence as the fusion tag for TcCAL1 to properly differentiate the recombinant from the endogenous protein in transgenic parasites. Although it cannot be completely ruled out, it seems unlikely that the presence of this fusion tag has exerted both beneficial and harmful phenotypes for *T. cruzi*, on the basis of the different parameters analyzed in this work.

The key early events in the invasion of mammalian cells by *T. cruzi* involve the recognition and adhesion to the target-plasma membrane, the parasite internalization and maturation inside a recently-assembled parasitophorous vacuole, and the release from this organelle-like-structure to differentiate into replicative amastigotes in the host cytosol (Andrews and Colli, 1982; Maeda et al., 2012; Rodríguez-Bejarano et al., 2021). Prior to cell invasion, the interaction of membrane surface molecules in *T. cruzi* and their ligands expressed in the host extracellular matrix initiates bidirectional signaling cascades which include Ca^2+^mobilization in the parasite (Manchola Varón et al., 2021). Then, *T. cruzi* harnesses several mechanisms for its internalization into the host cell, including lysosome-dependent exocytosis, endocytosis, or phagocytosis (Andrade and Andrews, 2004; Barrias et al., 2013; Batista et al., 2020). All these mechanisms depend on the developmental stage and/or the parasite strain and the host cell/tissue type. Some virulence factors have been identified for metacyclic trypomastigotes, such as the surface glycoprotein gp82, which mediates Ca^2+^-dependent entrance into the host cell (Mortara et al., 2005; Yoshida and Cortez, 2008). In light of the above findings, we envisage a scenario in which *T. cruzi* virulence is fostered by some beneficial function induced by TcCAL1-6xHis instead of cytosolic Ca^2+^ sequestration. In fact, more virulent strains trigger higher Ca^2+^ concentrations during their infection process (Ruiz et al., 1998; Epting et al., 2010). Would it be possible that TcCAL1 binds and/or activates key players in the invasion process? Could this protein be released outside the parasite, promoting the recognition, adhesion, or internalization, directly or indirectly into the host cell? Without doubt, the identification of the associated proteins or complexes, if any, and the localization of TcCAL1 during the invasion process could provide some clues to answer these questions. In this regard, Jesus et al. (2017) found TcCAL1 associated with parasite chromatin by proteomic tools. Although the authors pointed out that a mislocation due to contamination cannot be completely excluded, TcCAL1 was detected in two of the three extraction assays, being one of the most abundant proteins associated with chromatin. One interesting issue to be addressed is whether endogenous or recombinant TcCAL1 could regulate transcription, leading both to favor parasite virulence.

Finally, we observed a slight difference between the number of intracellular TcCAL1-6xHis-expressing amastigotes per Vero cell and their controls, which could correlate to some extent with the amastigote rates (**Figure 5A**). Although axenic and intracellular amastigotes exhibit differential features (Silva et al., 1998; Alcantara et al., 2021), the activity of phosphoinositide phospholipase C is Ca^2+^-dependent, an enzyme that is upregulated during differentiation from trypomastigotes into amastigotes (Furuya et al., 2000). However, there are no studies measuring the Ca^2+^ levels during this process. The role of TcCAL1 and its interaction with Ca^2+^ during amastigogenesis, and parasite attachment and internalization into the host cell is a current matter of investigation in our laboratory. In summary, our work strengthens the relevance of characterization the functions of hypothetical proteins to unravel *T. cruzi* biology.

## Data Availability Statement

The datasets presented in this study can be found in online repositories. The names of the repository/repositories and accession number(s) can be found below: https://tritrypdb.org/tritrypdb/app/workspace/strategies/import/6f350a00e98b079b. All other data in the study are available on request from the corresponding author.

## Ethics Statement

The animal study was reviewed and approved by the Institutional Commission for the Care and Use of Laboratory Animals (CICUAL) of University of Buenos Aires.

## Author Contributions

JRD and MP designed the study. JRD, JG, CAS, and MP performed the experiments and statistical analysis. MP supervised the study. KG and MP acquired funding. JRD and JG performed the artwork. JRD and MP wrote the original manuscript. JRD, JG, CAS, KG, and MP revised and edited the manuscript. JRD, KG, and MP reviewed and edited the final manuscript. All authors listed have made a substantial, direct, and intellectual contribution to the work and approved it for publication.

## Funding

This research was supported by grants from the Consejo Nacional de Investigaciones Científicas y Técnicas (CONICET; PIP No. 112-2015010-0937) and the Agencia Nacional de Promoción Científica y Tecnológica, Argentina (ANPCyT; PICT No. 2016-1028).

## Conflict of Interest

The authors declare that the research was conducted in the absence of any commercial or financial relationships that could be construed as a potential conflict of interest.

## Publisher’s Note

All claims expressed in this article are solely those of the authors and do not necessarily represent those of their affiliated organizations, or those of the publisher, the editors and the reviewers. Any product that may be evaluated in this article, or claim that may be made by its manufacturer, is not guaranteed or endorsed by the publisher.
